# *Lactobacillus paracasei* endocarditis of bioprosthetic aortic valve presenting with recurrent embolic strokes

**DOI:** 10.1099/acmi.0.000038

**Published:** 2019-07-26

**Authors:** Abdulfatah Osman, Michael Taipale, Mazen Najjar, Baraa Osman

**Affiliations:** ^1^ Department of Cardiovascular Medicine, 3452 Genesys Pkwy, Ascension Genesys Hospital, Grand Blanc, MI 48439, USA; ^2^ Division of Infectious Diseases, One Genesys Pkwy, Ascension Genesys Hospital, Grand Blanc, MI 48439, USA; ^3^ Touro College of Osteopathic Medicine, 60 Prospect Avenue, Middletown, NY 10940, USA

**Keywords:** *Lactobacillus paracasei*, endocarditis

## Abstract

**Introduction:**

*
Lactobacillus* prosthetic valve endocarditis is a rare infection caused by *Lactobacillus* bacteria. This bacterium is found in the normal flora of the human mouth, gastrointestinal tract and female genital tract. While there have been isolated cases of *Lactobacillus* bacteraemia and endocarditis, the infections are associated with comorbidities, immune deficiency, dental manipulation procedures and other medical history. This case of bioprosthetic valve endocarditis caused by *Lactobacillus paracasei* is unusual, as the patient was immune-competent and treated with pre-procedural antibiotics.

**Case:**

We present a case of a 65-year-old male who underwent a dental extraction. He presented after 3 months of fever, chills and fatigue. On initial presentation, blood cultures were positive for alpha-haemolytic streptococcus bacteraemia. He was treated with IV penicillin and underwent aortic valve replacement with a bioprosthetic valve and excision of the mitral vegetation with repair of the mitral valve. Two years later, he had a tooth extraction after being treated properly with antibiotics. Three months later he presented with difficulty speaking, left leg weakness and increased drooling. All testing was normal. Three months later he presented with left side lower extremity weakness and expressive aphasia. He was diagnosed with bioprosthetic aortic valve endocarditis and was treated with IV penicillin and gentamicin for 6 weeks and then switched to oral penicillin. He remained stable.

**Conclusions:**

*
L. paracasei* can potentially be a cause of complicated endocarditis in patients with prosthetic heart valves undergoing dental procedures. Timely culture-guided antibiotic therapy is critical and may obviate the need for valve surgery.

## Introduction

*Lactobacillus* bacteria are facultative anaerobic Gram-positive rods found in the normal flora of the human mouth, gastrointestinal tract and female genital tract. They are also abundant in lactate-containing dairy products and are used as a probiotic.

The genus has rarely been implicated in human disease. Isolated cases of *Lactobacillus* bacteraemia and endocarditis have been reported sporadically in the literature, but are still considered quite unusual. Clinical infections have predominantly been cited in association with severe comorbidities, immune deficiency, prolonged antibiotic use and recent surgeries, typically in the presence of structural heart disease [1].

Increased interest in the pathogenic potential of this bacterium has been generatedgiven the rising use of probiotics [2], the increasing population of immune-compromised patients and the widespread use of screening colonoscopy. *Lactobacillus* endocarditis has been reported in association with the above conditions in addition to dental manipulation procedures. Most reported cases have involved structurally abnormal native valves, with the most common pathogenic species being *Lactobacillus acidophilus, Lactobacillus casei* and *Lactobacillus rhamnosus *.

Herein, we report an unusual case of a bioprosthetic valve endocarditis caused by *Lactobacillus paracasei* in an immune-competent patient following a dental procedure that was appropriately treated by pre-procedural antibiotic prophylaxis.

## The case

Our patient is a 65-year-old male with a history of rheumatic fever, mitral valve prolapse and bicuspid aortic valve. He originally presented in June 2014 with 3 months of fever, chills and fatigue after undergoing a dental extraction. Blood cultures were positive for alpha-haemolytic streptococcus bacteraemia. Echocardiography confirmed diagnosis of aortic and mitral valve endocarditis. He was treated with IV penicillin and then underwent aortic valve replacement with a bioprosthetic valve and excision of the mitral vegetation with repair of the mitral valve. He had an uncomplicated post-operative course.

After an uneventful 2 years, he had a tooth extraction 30 min after receiving 2 g of oral amoxicillin. Three months later, the patient was admitted to the hospital for difficulty speaking, left leg weakness and increased drooling. After initial workup, his new neurological symptoms were concerning for an underlying cardio-embolic aetiology. A transesophageal echocardiogram (TEE) revealed a properly functioning bioprosthetic aortic valve with no apparent vegetations and no obvious source of cardiac emboli. He had no fever, chills or other constitutional symptoms. He also had a 30-day event monitor, which did not show any evidence of atrial fibrillation as a possible aetiology of his cerebrovascular accident.

Three months later, he once again presented to the emergency department complaining of new left side lower extremity weakness and expressive aphasia. He was diagnosed with recurrent transient ischemic attacks. A brain MRI showed evidence of prior bi-hemispheric infarcts suspected to be related to a central embolic source. He was again afebrile but had borderline leukocytosis. Initial blood cultures showed no growth at 48 h. Repeat transthoracic echo showed that his bioprosthetic aortic valve was functioning normally, with no evidence of vegetations. He was discharged home in stable condition.

**Fig. 1.**
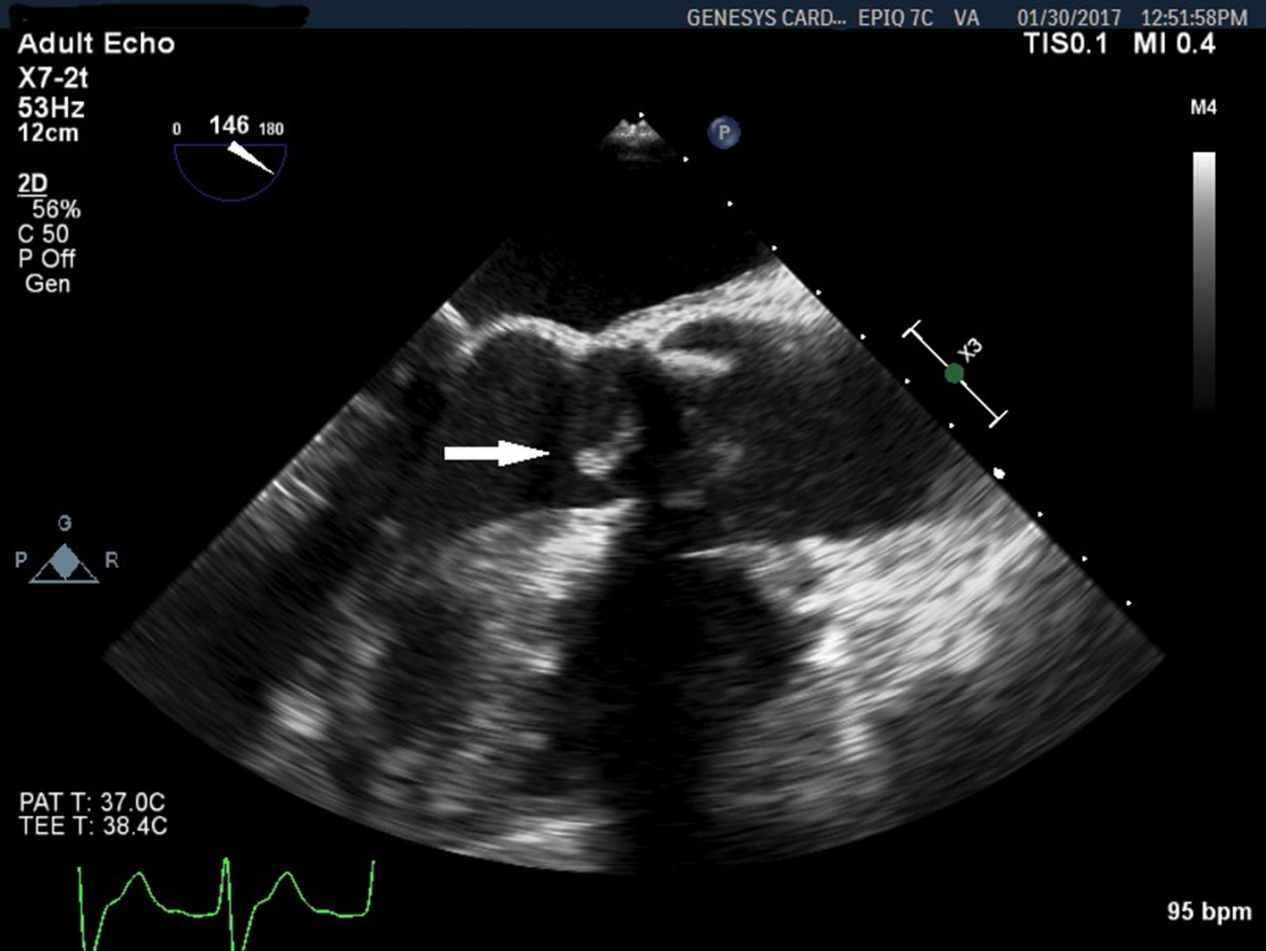
TEE 3 months after initial presentation shows a vegetation on the left ventricular side of the bioprosthetic aortic valve (arrow).

Three days later, the blood cultures turned positive in two out of three sets. ‘Gram-positive cocci in chains likely streptococci’ were initially reported. However, the bacterium was later confirmed to be a Gram-positive rod growing in both aerobic and anaerobic bottles. A repeat TEE was performed. It showed a highly mobile 1.2 cm vegetation on the bioprosthetic aortic valve (Fig. 1). Diagnosis of bioprosthetic aortic valve endocarditis was confirmed. The blood cultures were sent to a reference microbiology laboratory, where, using matrix-assisted laser desorption/ionization time-of-flight mass spectrometry (MALDI-TOF MS) [3], the organism was identified as *L. paracasei *. He was initially treated with IV vancomyocin and ceftriaxone. Once susceptibility results were obtained he was switched to IV penicillin and gentamicin for 6 weeks and then transitioned to oral penicillin, 500 mg four times daily. The patient responded favourably to antibiotic therapy and had no further neurological events.

Seven months later, he had a follow-up TEE, which showed complete resolution of the aortic valve vegetation. The valve demonstrated normal function, with no evidence of aortic insufficiency. Oral penicillin was stopped. Follow-up blood cultures 2 weeks after discontinuation of all antibiotics remained negative after 5 days of incubation. Surveillance blood cultures performed 3, 6 and 12 months after cessation of antibiotics were all negative. Over that time, he remained clinically stable, with no plan for repeat aortic valve replacement.

## Discussion

Cases of *L. paracasei* infective endocarditis are exceedingly rare [4]. Previous case reports of endocarditis caused by this pathogen have mostly involved patients consuming probiotics, although a causal relationship has not been established. Our patient had no history of consuming probiotics. Furthermore, he did not have known immune compromising comorbidities, such as diabetes, renal failure or severe debilitating diseases. However, the presence of a bioprosthetic aortic valve was likely a risk factor for the infection.

The temporal relationship to the dental procedure strongly suggests that *L. paracasei* bacteraemia as a result of dental manipulation was the culprit for valve infection. This took place despite strict compliance to appropriately dosed amoxicillin antibiotic prophylaxis prior to the procedure.

Antibiotic prophylaxis prior to dental procedures remains guideline-recommended in patients with prosthetic valves (class IIa, level of evidence C) [5]. However, the evidence supporting such a recommendation comes solely from observational studies. Therefore, whether antibiotic prophylaxis, even for high-risk patients, is actually effective in the prevention of endocarditis remains controversial. Our case highlights the need for careful follow-up of high-risk patients after potentially bacteraemic procedures, even if they received proper prophylaxis.

By clinical, echocardiographic and bacteriological findings, the diagnosis of prosthetic valve endocarditis was established in this case. Unlike in the vast majority of reported *Lactobacillus* endocarditis cases, our patient did not present with constitutional symptoms. Instead, he presented with recurrent cerebrovascular embolic events. Apart from one reported case complicated by thrombosis of the right posterior cerebral artery [6], to the best of our knowledge, our patient is the first reported case of *L. paracasei* endocarditis presenting as isolated recurrent ischemic strokes.

On the imaging side, this case is an example of the challenges frequently encountered in the diagnostic workup of prosthetic valve endocarditis. The initial transthoracic and transesophageal echoes did not show any vegetations, presumably due to shadowing artifacts by the valve struts. It is conceivable that a small vegetation was concealed by such artifacts during the initial imaging studies, and was only visible when it grew larger, as demonstrated on the follow-up transesophageal echo (Fig. 1). Moreover, no valvular regurgitation was noted during the initial or subsequent echo studies. These observations are consistent with the known insidious and less aggressive course of these unusual valvular infections [2]. They also highlight the need to maintain a high index of suspicion and keep a low threshold for repeating imaging and microbiological studies in pursuing the diagnosis.

Based on microbiological data and experience from prior reported cases of *Lactobacillus* endocarditis, penicillin and aminoglycosides remain the recommended empirical therapy [7]. Subsequent therapy should be guided by *in vitro* susceptibility testing. Such an approach has reduced mortality and resulted in good outcomes, as demonstrated by more recent case series and multivariate analysis [8, 9].

Our patient responded well to the penicillin and gentamicin regimen. Subsequent susceptibility testing confirmed sensitivity to penicillin. Provided there was a favourable clinical and echocardiographic response to medical therapy, high-risk repeat valve surgery was not felt to be necessary.

Despite it being a commensal organism, *Lactobacillus* pathogenicity has been well documented in the literature [9]. Experimental animal model studies have demonstrated the ability of some *Lactobacillus* isolates to adhere to connective tissue matrix proteins, which may impart a degree of virulence by promoting adherence to abnormal valve tissue. Such infectivity, however, was significantly inferior to that of more virulent organisms, such as staphylococcus or streptococcus [10]. Other models have shown different virulence factors for some *Lactobacillus* strains, such as platelet aggregation [11] and production of enzymes, which help with colonization and survival of the bacteria [12].

Therefore, *Lactobacillus* remains an important consideration in the differential diagnosis of initially culture-negative endocarditis given its known fastidious and slow growth properties in culture. Multiple blood cultures and extended incubation periods are often needed. Further, demonstration of positive blood culture should prompt screening for endocarditis by appropriate imaging techniques.

The favourable health benefits of probiotic micro-organisms have been widely studied and are well documented. These include promoting healthier gut flora, stimulating humoral immune responses and colorectal tumour cell suppression, among others [13]. Whether the pathogenicity of the otherwise commensal *Lactobacilli* is the result of host susceptibility factors or acquired virulent traits (for example due to interactions with other gut bacteria) remains unclear. Further understanding of these complex relationships is pertinent to the use of probiotics in vulnerable populations. Although the question of consumption of probiotics is reasonable to ask in any case of *L* *actobacillus* bacteraemia, the lack of consistent association, as demonstrated by our case, supports the need for further research in this field to investigate any causal relationship if present.

### Conclusion

*Lactobacillus paracasei* is a rare, but important, cause of potentially complicated endocarditis in patients with prosthetic heart valves undergoing dental procedures, regardless of antibiotic prophylaxis use. Timely culture-guided antibiotic therapy is critical and, at least in some cases, may obviate the need for valve surgery.

## Methods

This study is a case report. It describes the clinical course of a patient starting in June 2014 and lasting until February 2019. All clinical evaluation, diagnostic testing, imaging studies and treatments were delivered at Ascension Genesys Hospital, Grand Blanc, Michigan, United States of America.

## References

[1] Cannon JP, Lee TA, Bolanos JT, Danziger LH (2005). Pathogenic relevance of Lactobacillus: a retrospective review of over 200 cases. Eur J Clin Microbiol Infect Dis.

[2] Franko B, Vaillant M, Recule C, Vautrin E, Brion JP (2013). Lactobacillus paracasei endocarditis in a consumer of probiotics. Med Mal Infect.

[3] Oakey HJ, Harty DW, Knox KW (1995). Enzyme production by lactobacilli and the potential link with infective endocarditis. J Appl Bacteriol.

[4] Singhal N, Kumar M, Kanaujia PK, Virdi JS (2015). MALDI-TOF mass spectrometry: an emerging technology for microbial identification and diagnosis. Front Microbiol.

[5] Hoen B, Alla F, Selton-Suty C, Béguinot I, Bouvet A (2002). Changing profile of infective endocarditis: results of a 1-year survey in France. JAMA.

[6] Nishimura RA, Otto CM, Bonow RO, Carabello BA, ErwinIII JP (2017). AHA/ACC focused update of the 2014 AHA/ACC guideline for the management of patients with valvular heart disease. J Am Coll Cardiol.

[7] Dupont B, Lapresle Cl (1977). [Subacute bacterial endocarditis due to lactobacillus (author's transl)]. Nouv Presse Med.

[8] Griffiths JK, Daly JS, Doge RA (1992). Two cases of endocarditis due to Lactobacillus species: antimicrobial susceptibility, review and discussion of therapy. Clin Infect Dis.

[9] Salminen MK, Rautelin H, Tynkkynen S, Poussa T, Saxelin M (2004). *Lactobacillus* bacteremia, clinical significance, and patient outcome, with special focus on probiotic *L.* rhamnosus GG. Clin Infect Dis.

[10] Salvana EM, Frank M (2006). Lactobacillus endocarditis: case report and review of cases reported since 1992. J Infect.

[11] Vankerckhoven V, Moreillon P, Piu S, Giddey M, Huys G (2007). Infectivity of *Lactobacillus rhamnosus* and *Lactobacillus paracasei* isolates in a rat model of experimental endocarditis. J Med Microbiol.

[12] Harty DW, Patrikakis M, Hume EB, Oakey HJ, Knox KW (1993). The aggregation of human platelets by *Lactobacillus* species. J Gen Microbiol.

[13] Soltan Dallal MM, Mojarrad M, Baghbani F, Raoofian R, Mardaneh J (2015). Effects of probiotic *Lactobacillus acidophilus* and *Lactobacillus casei* on colorectal tumor cells activity (CaCo-2). Arch Iran Med.

